# MHC Class I Bound to an Immunodominant *Theileria parva* Epitope Demonstrates Unconventional Presentation to T Cell Receptors

**DOI:** 10.1371/journal.ppat.1001149

**Published:** 2010-10-14

**Authors:** Isabel K. Macdonald, Maria Harkiolaki, Lawrence Hunt, Timothy Connelley, A. Victoria Carroll, Niall D. MacHugh, Simon P. Graham, E. Yvonne Jones, W. Ivan Morrison, Darren R. Flower, Shirley A. Ellis

**Affiliations:** 1 The Jenner Institute, University of Oxford, Compton, Berkshire, United Kingdom; 2 Division of Structural Biology, Wellcome Trust Centre for Human Genetics, University of Oxford, Oxford, United Kingdom; 3 Institute for Animal Health, Compton, Berkshire, United Kingdom; 4 The Roslin Institute, Royal (Dick) School of Veterinary Sciences, University of Edinburgh, Edinburgh, United Kingdom; 5 Veterinary Laboratories Agency, Addlestone, Surrey, United Kingdom; Trudeau Institute, United States of America

## Abstract

T cell receptor (TCR) recognition of peptide-MHC class I (pMHC) complexes is a crucial event in the adaptive immune response to pathogens. Peptide epitopes often display a strong dominance hierarchy, resulting in focusing of the response on a limited number of the most dominant epitopes. Such T cell responses may be additionally restricted by particular MHC alleles in preference to others. We have studied this poorly understood phenomenon using *Theileria parva*, a protozoan parasite that causes an often fatal lymphoproliferative disease in cattle. Despite its antigenic complexity, CD8^+^ T cell responses induced by infection with the parasite show profound immunodominance, as exemplified by the Tp1_214–224_ epitope presented by the common and functionally important MHC class I allele N*01301. We present a high-resolution crystal structure of this pMHC complex, demonstrating that the peptide is presented in a distinctive raised conformation. Functional studies using CD8^+^ T cell clones show that this impacts significantly on TCR recognition. The unconventional structure is generated by a hydrophobic ridge within the MHC peptide binding groove, found in a set of cattle MHC alleles. Extremely rare in all other species, this feature is seen in a small group of mouse MHC class I molecules. The data generated in this analysis contribute to our understanding of the structural basis for T cell-dependent immune responses, providing insight into what determines a highly immunogenic p-MHC complex, and hence can be of value in prediction of antigenic epitopes and vaccine design.

## Author Summary

CD8^+^ T cells are essential for host defense to many intracellular pathogens. The CD8^+^ T cell receptor (TCR) detects small peptide fragments presented at the surface of infected cells. These peptide epitopes are bound by major histocompatibility complex (MHC) molecules. Such T cell responses are often inexplicably focused on a very small number of epitopes, even in response to large and complex pathogens. These epitopes are usually presented by specific MHC molecules. This phenomenon is known as immunodominance. An increased understanding of the factors involved in immunodominance would increase our ability to design more effective vaccines against a range of infectious pathogens. Our study focused on an economically important protozoan parasite of cattle, *Theileria parva*. We generated crystals of MHC molecules bound to an immunodominant *T. parva* epitope. Analysis of the crystal structure demonstrated that the peptide was held in a highly unusual conformation, which was shown to impact significantly on TCR recognition, based on studies of specific CD8^+^ T cell clones. These data will aid our understanding of the structural basis for T cell immunity and will ultimately contribute to development of disease control strategies.

## Introduction

T cells constitute a key component of the adaptive immune system, allowing recognition of virtually any pathogen that may infect the host. Immunity against many intracellular pathogens is governed by CD8^+^ T cells and major histocompatibility complex (MHC) class I molecules, which bind antigenic peptides within a groove formed by their alpha 1 and alpha 2 extracellular domains [1]. Recognition by the clonotypic T cell receptor (TCR) of the peptide-MHC (pMHC) complex is a critical step in this process, leading to a variety of responses aimed at elimination of the pathogen [2]. These processes have been largely characterised in humans and mice, and while the same immune system components are found in cattle, an economically important livestock species, less is known of the detailed cellular and molecular interactions in this species.

MHC class I genes are highly polymorphic and it is this feature that determines individual peptide binding characteristics of class I molecules [3]. Cattle have at least 6 classical MHC class I genes [4] (contrasting with 3 in human), and variable haplotype structures, with usually one, 2 or 3 of the genes present and expressed, in a variety of combinations [5]. We have previously identified the majority of MHC class I alleles (www.ebi.ac.uk/ipd/mhc/bola) present in the Holstein or Friesian breed (*Bos taurus*), which represents by far the most abundant breed of dairy cattle throughout Europe and North America and is increasingly being used to improve milk production in developing countries. MHC class I allelic diversity in the Holstein/Friesian cattle population is relatively low in comparison to similarly large human populations, for example Caucasian; this probably reflects a combination of founder effect and/or recent strong selection for production traits (Ellis, manuscript submitted).

The structural basis for peptide binding to MHC class I molecules has been extensively investigated in humans and mice [6], [7], less so for other species [8], [9]. The N and C termini of bound peptides interact with invariant class I residues at either end of the peptide-binding groove. Peptides are most often between 8 and 10 residues in length, and although longer exceptions that adopt a bulged conformation have been reported in human [10], rat [9] and mouse [11] these have generally been considered very unusual. The peptide specificity exhibited by MHC molecules is dependent on the entire sequence of binding peptides, but is dominated by several key interactions made by so-called anchor residues. These define a peptide binding ‘motif’ for a particular MHC allele which usually consists of two or more anchor residues, such that in all peptides that will bind the allele these two residues will be relatively invariant or have conserved properties. Structural studies have revealed that the side chains of the peptide anchors interact with polymorphic residues in a series of pockets in the groove; these define the binding specificity of the allele [12]. Many peptide binding motifs have been described in human and mouse, while in cattle peptide binding properties have been investigated for only a small number of MHC class I alleles [13], [14], and data are insufficiently detailed to allow meaningful comparison between species.

A characteristic feature of CD8^+^ T cell responses to many pathogens is that the peptide epitopes display a strong dominance hierarchy, resulting in focusing of the response on a limited number of the most dominant epitopes [15]. Such T cell responses may be additionally restricted by particular MHC alleles in preference to others. This phenomenon impacts substantially on our ability to design and implement improved vaccines against a range of pathogens, yet is still poorly understood. We wished to investigate the basis for this phenomenon using the example of *Theileria parva*, a complex protozoan parasite for which the cattle CD8^+^ T cell response has been extensively studied and characterized.

Several CD8^+^ T cell epitopes in *T. parva* have been identified recently [16], [17], [18]. Graham et al [18] investigated recognition of synthetic peptides by antigen-specific CD8^+^ T cells and showed that out of 9 epitopes derived from 6 different *T. parva* antigens 3 were 11 amino acids long. One of these (Tp1_214–224_) was shown to be presented by the cattle MHC class I allele N*01301. This allele is expressed alone on the haplotype designated A18, which is common in the British Friesian breed. Although this is not the only single gene class I MHC haplotype in cattle, it remains an unusual phenomenon in mammals (where 2 or 3 class I genes are generally expressed), but is commonly seen in non-mammalian vertebrates [19]. MacHugh et al [20] showed that in *T. parva*-immune A18-homozygous animals, over 75% of responding T cells are specific for the Tp1_214–224_ 11mer. Animals heterozygous for A18 also consistently respond to this epitope presented by N*01301 [18], [20]. Dominant CD8^+^ T cell responses restricted by this allele have in addition been reported for respiratory syncytial virus [21] and foot and mouth disease virus [22].

Determination of the structural and biophysical characteristics of pMHC interactions can lead to a better understanding of T cell recognition and T cell-dependent immune responses. Unique features of the MHC in cattle [23], and the pressing requirement for improved vaccines and other disease control measures in livestock [24], mean that increasing our knowledge in this area will not only shed light on evolution of the mammalian immune system but will also be of practical benefit. Here we describe the peptide binding properties of the common and functionally important cattle MHC class I allele, N*01301. We also describe, at atomic resolution, the crystal structure of N*01301 bound to an immunodominant 11mer *Theileria parva*-derived peptide, Tp1_214–224_. This analysis provides important clues regarding the features that determine immunodominance of this pMHC structure. As far as we are aware, this is the first description of an MHC structure from a commercially important mammalian livestock species.

## Results

### Peptide Binding Characteristics of N*01301

**Figure 1:**
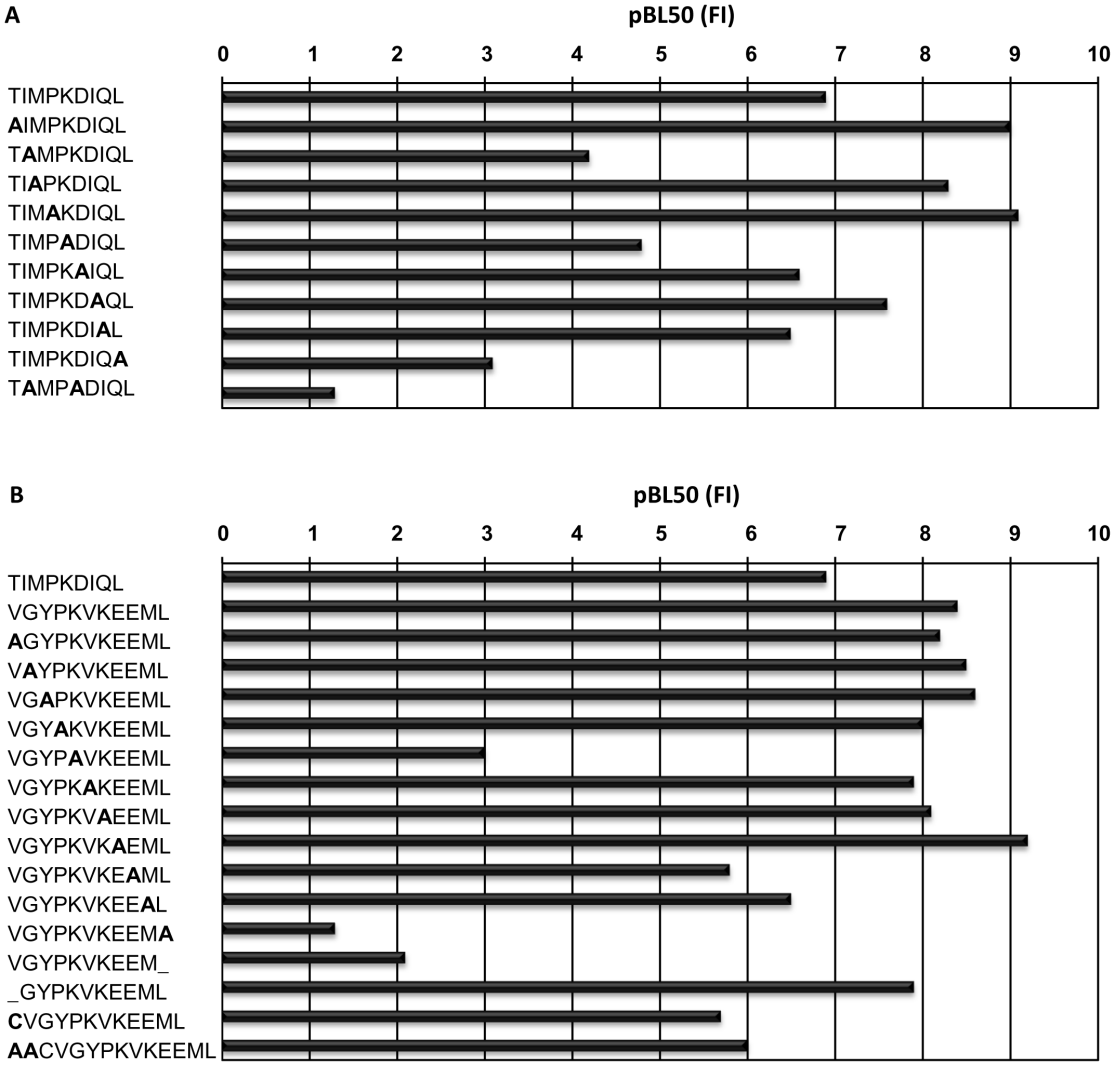
Peptide binding to N*01301. pBL_50_ values obtained with N*01301 using an RMA-S stabilization assay, (see Materials and Methods for explanation), (A) for the reference peptide (TIMPKDIQL) and Ala-substituted peptides and (B) forTp1_214–224_ (VGYPKVKEEML) and Ala- substituted peptides.

A 9mer self-peptide (TIMPKDIQL) shown to have a high binding affinity to N*01301 was used throughout as a positive control in flow-based MHC stabilization assays (see Materials and Methods for details). In order to systematically explore the relative contribution to binding affinity made by amino acids at each position, a sequential alanine-scan of the self-peptide was made (Fig. 1A). Dose/FI curves were created for each peptide (using peptides at concentrations between 0.1 nM and 1 mM) and presented as semi-logarithmic plots as in previous studies ([25], supporting information (SI) Fig. S1). The high binders have low half-maximal binding level (BL_50_) values (high pBL_50_) and the low binders have high BL_50_ values (low pBL_50_). Peptides that did not reach 50% of the binding level of the reference peptide were considered non-binders. The pBL_50_ ranges are: for low binding <4 (BL_50_ >10^−5^), for intermediate binding between 4 and 5, and for high binding >5 (BL_50_ <10^−5^). A parallel study was performed using the 11mer *T. parva*-derived epitope Tp1_214–224_ VGYPKVKEEML ([17], Fig. 1B).

N*01301 binding affinities for most of the Ala-substituted versions of the 9mer self-peptide demonstrated only minor differences, with three exceptions. The conversion to Ala of Leu at the C-terminal position, Ile at position 2 (P2) or Lys at position 5 resulted in a substantial loss of binding, while a peptide with Ala substitutions at positions 2 and 5 showed an even greater loss of binding. Peptides substituted at positions 2 and 9 or 5 and 9 were non-binders (or binding was <50% of the reference peptide). These results suggest that Ile, Lys and Leu are anchors at positions 2, 5 and 9 respectively. These findings are in partial agreement with previous work on the N*01301 allele which identified Ile as an auxiliary anchor at position 2, and Lys as a preferred residue at position 5 [14].

N*01301 binding affinities for most of the Ala-substituted versions of the 11mer Tp1_214–224_ peptide also demonstrated only a few differences (Fig. 1B). Similar to the self peptide, the Ala-substitution or removal of the C-terminal Leu resulted in the largest loss of peptide binding for a single substitution/deletion. Ala-substitution of Lys at position 5 also resulted in a substantial loss in binding. In contrast to the 9mer peptide, substitution at position 2 of the 11mer, which in this case was Gly, had no effect. A peptide with positions 5, 7 and 11 Ala-substituted was a non-binder. Peptides were also synthesized that probed how varying the length of Tp1_214–224_ would affect its ability to bind N*01301. Removing one residue from the N terminus of this peptide resulted in no appreciable difference in binding, which suggests this shorter peptide binds in a similar fashion to Tp1_214–224_. However this 10mer was not recognized by CD8^+^ T cells, suggesting that removal of residue 1 alters the conformation of the bound peptide [18]. This is therefore only partially consistent with observations made for HLA-A*0201 crystal structures [26], that revealed octamer and nonamer versions of a peptide binding with the same conformation, due to replacement of the missing N-terminal residue with water molecules.

Additions to the N terminus, of either a Cys which is the naturally occurring amino acid within the *T. parva* protein, or a Cys and two Ala also had little effect. It is possible that an altered N-terminal residue would occupy the same pocket as the Val found in Tp1_214–224_, while additional residues are likely to extend out of the binding groove, as reported for a mouse H-2K^b^-bound peptide [27]. These findings suggest that while the C-terminal Leu is the most crucial residue ensuring peptide binding to the N*01301 allele, amino acid changes at other positions can also substantially reduce binding, even if the Leu is present. The relative importance of these other positions is likely to differ depending on the length of the peptide and its position in the peptide binding groove.

### Overview of Cattle MHC Class I N*01301 Structure

**Figure 2:**
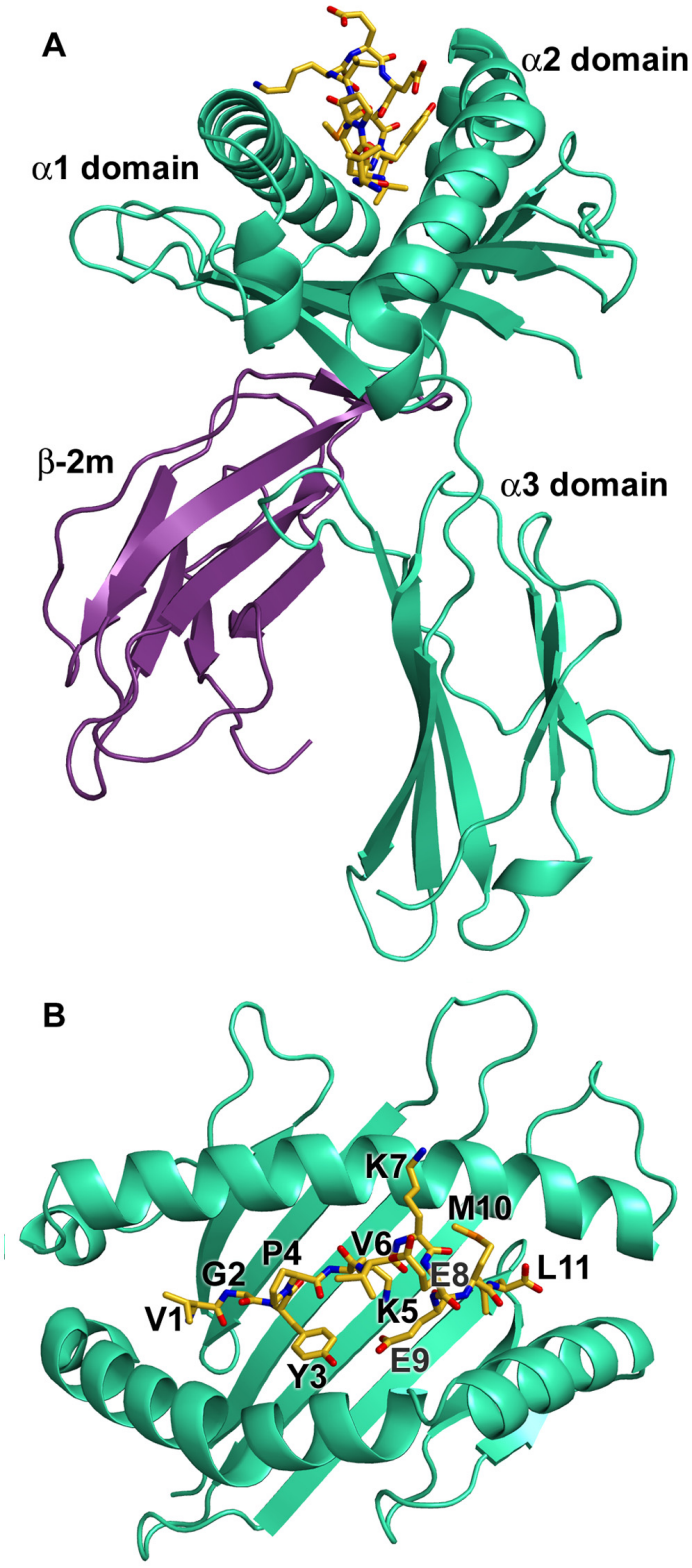
Overview of the structure of N*01301. (A) The heavy chain is shown in cyan, β_2_m in purple, and the peptide in yellow (B) Top view of the binding platform showing the bound peptide Tp1_214–224_ in stick format with residues labeled.

The N*01301- Tp1_214–224_ complex yielded well-ordered crystals, high resolution x-ray diffraction data were collected and the structure determined by molecular replacement (see Materials and Methods and Table S1 for details). The overall structure of N*01301 resembles that of other mammalian MHC class I molecules ([3], Fig 2A). Comparison with pMHC structures from other species indicates that the orientation of the N*01301 α3 domain relative to the rest of the MHC class I molecule lies within the range reported for mouse pMHCs [11], [27], but is distinct compared to the average position of human α3 domains, with a 5–7° clockwise rotation relative to the molecular long axis. However, the N*01301 α3 domain does not show the large (14–29°) differences in orientation found between the human molecules and chicken BF2*2101 [8]. This particular orientation of the α3 domain may have some effect on biophysical characteristics of the species-specific interactions of the cattle α3 domain with other molecules in the immunological synapse.

**Figure 3:**
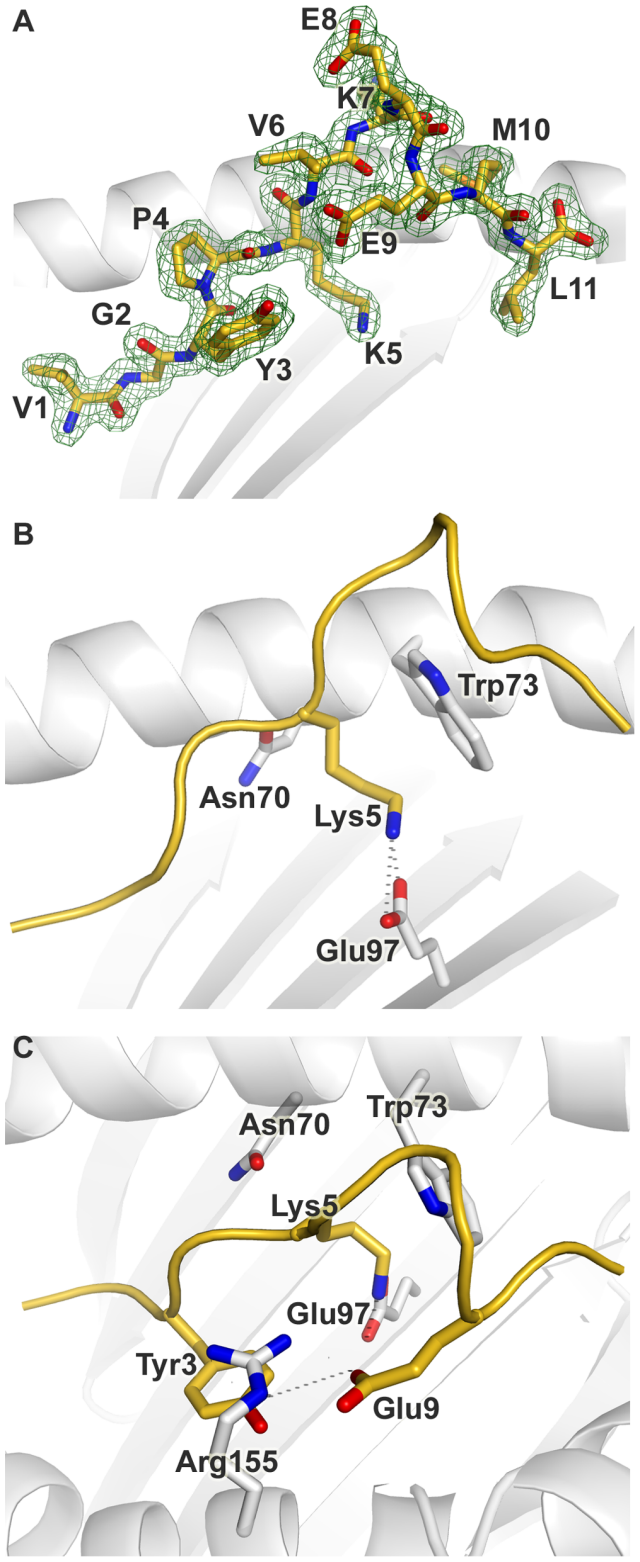
Conformation of the bound peptide. Conformation of the bound peptide Tp1_214–224_ shown from the α2 helix side of the binding groove, unless stated otherwise. (A) The corresponding *2F_o_-F_c_* electron density is shown in mesh format. The peptide is in stick format with each amino acid labeled (B) View onto the binding groove (as in Fig. 2B) additionally showing Trp3 and Glu9 of Tp1_214–224_, and Arg155 of N*01301. Hydrogen bonds are denoted by dotted grey lines (C) The peptide backbone is shown as a cartoon ribbon in gold. The Lys at position 5 of Tp1_214–224_ and the Asn, Glu and Trp at positions 70, 97 and 73 respectively of N*01301 are shown in stick format.

The N (position 1, P1) and C (PΩ) termini of the Tp1_214–224_ peptide conform to the canonical pMHC class I binding mode, anchored in the A and F pockets respectively of the N*01301 binding groove (Fig. 2B, Fig. S2). The P1-Cα/PΩ-Cα distance of 22Å is typical of pMHC complexes [28] and, as reported for other 11mer peptide complexes [10], requires a substantial portion of the peptide to bulge out of the binding groove (Fig. 3A). In the N*01301- Tp1_214–224_ complex the bulged conformation is tethered to the binding groove at P5 and P9 with residues P6-P8 prominently exposed at the pMHC surface (Fig. 3A). The peptide conformation appears to be relatively rigid (the electron density is well-defined for the full-length of the peptide, Fig. 3A) and is not the result of stabilizing interactions with neighboring molecules in the crystal (the peptide does not contribute to any major crystal-packing contacts). This is in contrast to the flexibility in peptide conformation observed for some of the previously determined highly bulged pMHC structures [29], [30], [31].

The overall architecture of the N*01301 binding groove and the peptide main chain conformation is typical of pMHC complexes for positions 1–3 (P1-P3). At P2 the conformation is stabilized by van der Waals (VDW) contacts with Tyr7 in pocket A and hydrogen bonding with Glu63 in pocket B (Fig. S2). In the absence of a P2 side chain (P2 is a Gly in Tp1_214–224_), pocket B is occupied by three bound water molecules. Displacement of one or more of these water molecules could provide sufficient space for pocket B to accommodate an alanine side chain, consistent with our Ala-substituted Tp1_214–224_ binding data (Fig. 1B). The P3 main chain is tethered by an interaction with Tyr99 (Fig. S2), while the P3 side chain (tyrosine) is anchored in pocket D by hydrophobic stacking with Arg155 (Fig. 3C). Arg155 also interacts with the peptide through hydrogen bonding to the main chain at P4 and the side chain of the Glu P9 residue.

Electrostatic potential calculations show that the central region of the N*01301 binding groove displays strong electronegative potential (Fig. S4), augmented by Glu114 which is involved in a H-bond network with Glu97 as well as with water molecules present in pockets C and D. Glu97 points up from the floor of the groove to hydrogen bond to the Lys side chain of P5 (Fig. 3B). This interaction results in P5 extending deep into the binding groove, the side chain conformation further stabilized by hydrophobic stacking with Trp73. Thus, consistent with our peptide binding data, Lys at P5 acts as a major anchor residue. The main chain conformation of the P6-P8 bulge is stabilized by partial hydrogen bonds of the P6 and P9 carbonyl oxygens to the Nε of Trp73 as well as intra-peptide bonds between the same carbonyl atoms and the main chain nitrogen atoms at P7 and P8. The bulky Trp73 residue thus appears to play a defining role in the location and stabilization of the P6-P9 bulge conformation (Fig. 3B and C). The peptide main chain dips back into the binding groove at P9, the Glu side chain pointing back along the groove to make a hydrogen bond to Arg155. Finally, the Leu side chain at PΩ is anchored in pocket F by hydrophobic interactions with residues Tyr116, Met147, Ala77, Leu81 and Tyr123 (Fig. S2).

Overall the binding of Tp1_214–224_ to N*01301 results in an interface of 1040 Å^2^, one of the most extensive interface areas for canonical pMHC complex structures reported to date (Table S2). Most of the pMHC interfaces for human alleles average no more than 900 Å ^2^ and structures for other species reveal that only mouse MHC class I molecules engage consistently through interfaces nearing the N*01301 value. While forming this extensive interface, the Tp1_214–224_ peptide also presents a substantial number of surface-exposed residues for T cell receptor (TCR) recognition, namely P1 (Val), P4 (Pro), P6 (Val), P7 (Lys), P8 (Glu) and P10 (Met), (Fig. 3A, Fig. S5).

### Comparison to Other MHC Class I Structures

**Figure 4:**
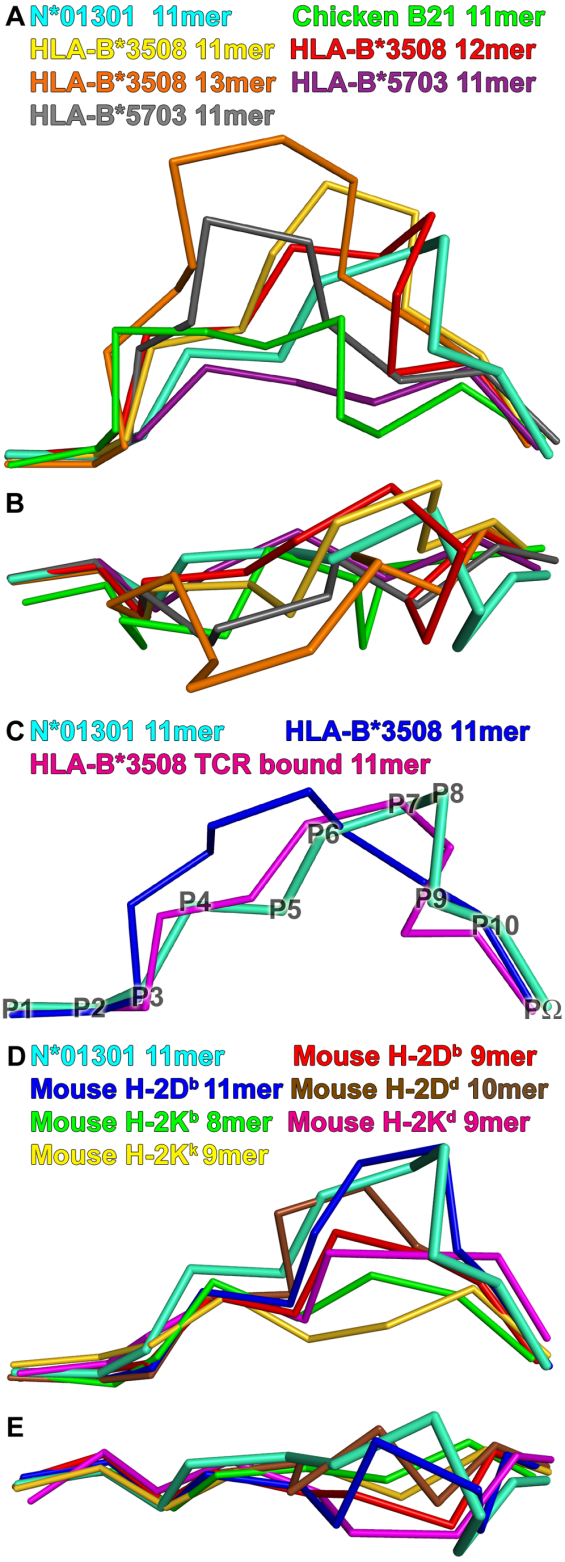
Comparison of structures. Peptide Tp1_214–224_ bound to N*01301 (cyan), compared to peptides in other mammalian pMHC structures. View shows the Cα-traces of the bound peptide from the α2 helix side of the binding groove, unless stated otherwise. (A) Long peptide comparison: 11mer peptide (green, PDB accession code 3BEV) bound to chicken B21; 11mer (gold, PBD code 2FZ3), 12mer (red, PBD code 3BW9) and 13mer (orange, PBD code 1ZHL) peptides bound to HLA-B*3508; 11mer peptides (purple, PBD code 2BVQ; grey, PBD code 2BVO) bound to HLA-B*5703. (B) Long peptide comparison: view onto the binding groove of Fig. 4A. (C) Long peptide comparison: 11mer peptide bound to HLA-B*3508 (blue, PDB code 1ZSD), and its conformation change upon TCR binding (pink, PDB code 2NX5). Peptide Cα backbone positions are denoted by P1-PΩ for Tp1_214–224_. (D) Mouse comparison: 9mer peptide (red, PBD code 1CE6) and 11mer peptide (blue, PBD code 1JPF) bound to H-2D^b^; 10mer peptide (brown, PBD code 1BII) bound to H-2D^d^; 8mer peptide (green, PBD code 1FO0) bound to H-2K^b^; 9mer peptide (pink, PBD code 1VGK) bound to H-2K^d^; 9mer peptide (yellow, PBD code 1ZT1) bound to H-2K^k^. (E) Mouse comparison: view onto the binding groove of Fig. 4D.

Least squares superpositions of the binding groove of cattle N*01301 with all available pMHC crystal structures allowed a comparison of key features. Figure 4 (A, B) shows a comparative overlay of the N*01301 bound peptide Tp1_214–224_ with a number of representative longer (>10mer) peptides from other pMHC structures. Structures reported to have flexible or multiple peptide conformations were not included [29], [30], [31]. All of the peptides have standard N- and C-terminal positions within the binding groove. The human alleles HLA-B*35 and B*57 bind longer peptides up to 14 residues in length [29]. Examples are shown in Figs 4A and B together with an 11mer peptide bound to chicken BF2*2101 [8]. The canonical N- and C- terminal anchor positions can be spanned by 8mer and 9mer peptides in relatively extended conformations; longer peptides are accommodated by adopting either zig-zag conformations within the groove or bulging out of the groove (Fig. 4B). Tp1_214–224_ is distinctive in having the bulge, P6–P8, located nearer the C-terminal end of the binding groove than the other examples of bulged-type conformations.

There are many HLA-B*35 structures (*3501 and *3508) binding both short (9mer) and longer peptides (11–14mers); the latter for the most part bulge centrally. Only two examples of long peptides out of fifteen available structures display C-terminal bulging; both are bound to HLA-B*3508 (Fig. 4A and 4B: 11mer in yellow [32]; 12mer in red [30]). Of these the 12mer bulges out closer to the C terminal end of the B*3508 binding groove (P7–P10) than any other human class I-bound peptide, but this bulge is still somewhat less C-terminal than its counterpart in Tp1_214–224_ (PΩ-2 and PΩ-1 are surface exposed, respectively). In both the HLA-B*3508-11mer and -12mer structures the C-terminal bulge position results from P5 (Glu) acting as a secondary anchor, forming salt bridges to Arg97 and Arg156. The use of P5 as an anchor in Tp1_214–224_ also involves interaction with N*01301 residue 97 but in this case P5 is the positively charged residue Lys interacting with the negatively charged Glu97; there is no interaction with residue 156 (Phe), but there is instead an additional interaction with Trp73. Figure 4C shows the effect of TCR binding for a second HLA-B*3508-11mer complex [31]. The peptide in this case has adopted a ‘flattened’ conformation that bulges more towards the C terminus of the peptide binding groove compared to its non-TCR bound equivalent [28].

The above analysis highlights the contribution of Trp73 to two distinctive characteristics of the N*01301 groove: the preference for Lys as a P5 anchor residue and the main chain conformation of the C terminal (P6-PΩ) portion of the peptide. Trp73 stabilizes the P9 (PΩ-2) residue on the α2 helix side of the binding groove at a position distinct from those of PΩ-2 residues in human pMHC structures (Fig. 4B and Fig. S3). Tryptophan at position 73 is extremely uncommon. It does not occur in MHC class I sequences of any of the species included in the immunopolymorphism database (www.ebi.ac.uk/ipd/mhc) or in human, where this position is occupied almost exclusively by Ala or Thr (www.ebi.ac.uk/imgt/hla/). In cattle it occurs only in N*01301 and in 5 very closely related alleles, all encoded at the same locus (Table 1). In contrast Trp73 is relatively common in mouse MHC class I molecules, occurring in ∼25% of alleles including L^d^, L^q^, D^q^ and D^b^. In mouse it is always accompanied by Tyr at position 156, which together with Trp at 147 produces a hydrophobic ‘ridge’ that forces the bound peptide out of the groove [11]. A comparable ridge, formed by Tyr152, Trp70 and Tyr9 is seen in the rat RT1-A^a^ molecule [9]. Cattle N*01301 has Trp at 73 and Met at 147, which together would be predicted to form a similar feature, conserved in the alleles included in Table 1.

**Table 1:** Amino acid residues at positions 73 and 147 in 7 cattle MHC class I alleles.

Allele	73	147
N*01301	W	M
N*01302	W	M
N*01401	W	W
N*01402	K^1^	W
N*01501	W	M
N*01502	W	M
N*04101	W	M

1 BoLA N*01402, which has Lys in place of Trp at position 73, has only been recorded once (www.ebi.ac.uk/ipd/mhc/bola); Lys at position 73 has not been observed in any other cattle class I allele.

Figs 4D and E show peptides bound to several mouse MHC class I molecules. In H-2D^b^, which exhibits the hydrophobic ridge, P5 is a central anchor point as in the cattle structure. The side chain of the P5 residue, in this case an Asn, reaches deep into the binding groove and occupies the same position as the Tp1_214–224_ Lys, interacting with residues at the bottom of the groove. As a consequence of the smaller side chain, the P5 Cα backbone is shifted by 1.5 Å. Furthermore, the peptide main chain interacts with Trp73 in the same way, with its P6 and P9 carbonyl oxygens forming similar partial hydrogen bonds to the N_ε_ of Trp73. Thus the characteristics of the cattle N*01301 binding groove are most similar to those of the mouse MHC class I molecules, in particular H-2D^b^.

### Recognition of the N*01301-Tp1_214–224_ Structure by CD8^+^ T Cell Clones

**Figure 5:**
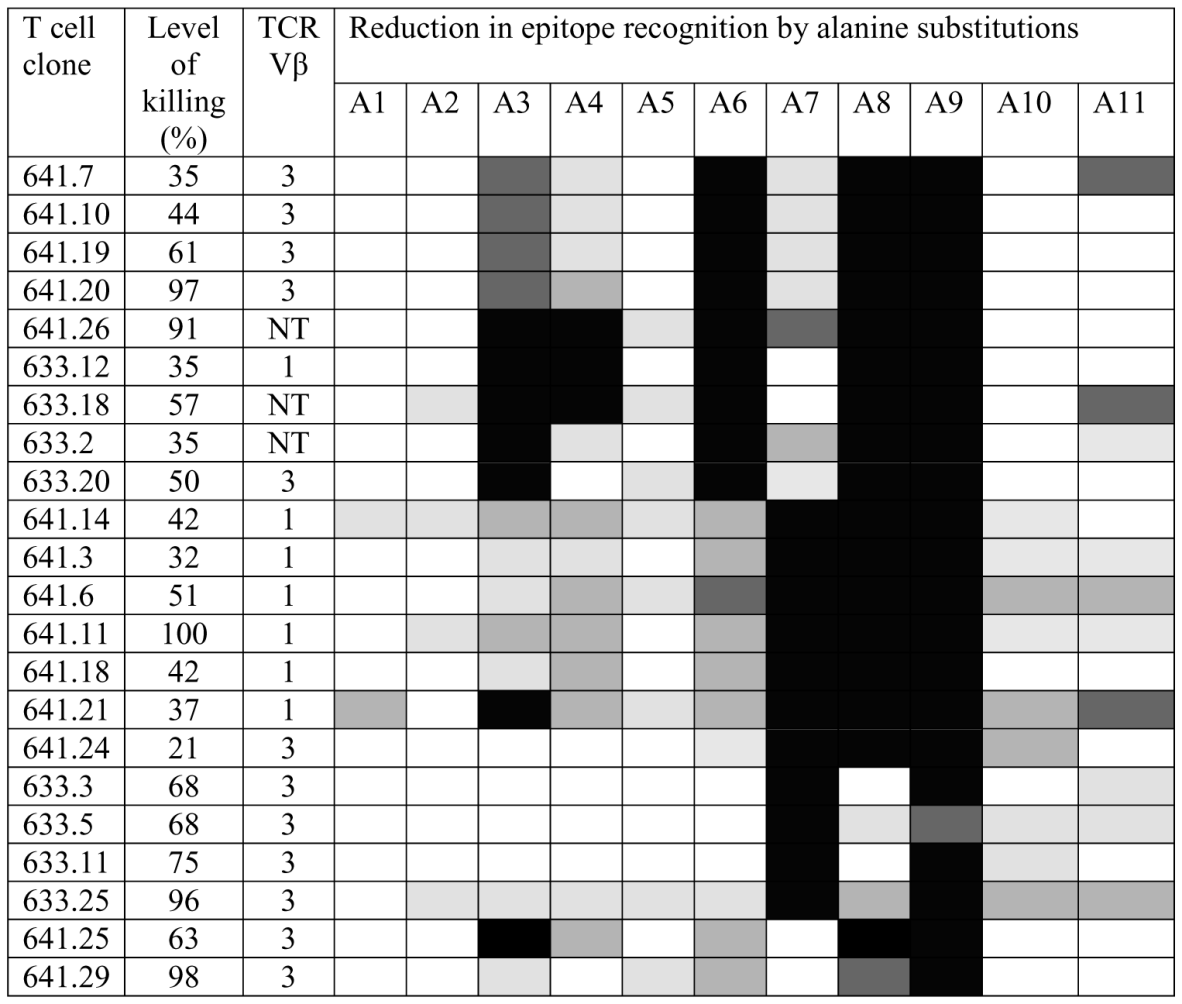
Effects of single alanine substitutions in the Tp1_214–224_ epitope on recognition by specific CD8^+^ T cell clones. T cell recognition was examined using a 4-hour ^111^Indium release cytotoxicity assay employing target cells incubated with 1 ug/ml of peptide. The maximum level of cytotoxicity obtained with the native Tp1_214–224_ peptide is shown for each clone. Killing of unpulsed target cells was <2% for all clones. The degree of reduction in killing obtained with each of the single Ala substituted peptides (A1–A11) compared to that obtained with the native Tp1_214–224_ peptide is represented as follows: 0–20% reduction (white); 21–40% reduction (pale grey); 41–60% reduction (medium grey); 61–80% reduction (dark grey); 81–100% reduction (black). The expressed TCR Vβ genes were identified using Vβ subfamily-specific PCR assays. NT = not tested.

Since the characteristics of the N*01301- Tp1_214–224_ structure (i.e. highly stable, large area exposed to TCR, unusual positioning of the peptide bulge) suggest it may have a major impact on TCR recognition, we performed functional studies to determine the fine specificity of T cell recognition. Twenty-two Tp1_214–224_-specific CD8^+^ T cell clones, derived from 2 T. *parva*-immune animals (641 and 633), were examined for their ability to recognize a series of Ala-substituted versions of Tp1_214–224_ (as detailed in Fig 1), using a cytotoxicity assay. All clones gave near maximal levels of killing of cells pulsed with the native peptide (ranging from 21% to 100% for different clones) down to a concentration of 10 pg/ml (example shown in Fig S6). The results obtained with the Ala-substituted peptides tested at 1 ng/ml (i.e. well above the minimal concentration required to obtain maximal killing), are summarized in Fig 5. The most striking finding was the abolition (or near abolition) of recognition by all T cell clones of 2 or more of the peptides substituted at positions 6–9. Based on major effects on recognition of peptides substituted at positions 6, 7 and 8, the 22 clones fell into 4 groups each with broadly similar specificity. Nine clones were profoundly influenced by residues 6 and 8, seven clones by residues 7 and 8, four clones by residue 7 and two clones by residue 8. Substitution of position 9 profoundly reduced recognition by all 22 clones. Substitutions at positions 3 and 4 also had substantial effects on recognition by some clones.

The evidence that residues 6–9 are involved in epitope recognition by all T cell clones indicates that the CD8^+^ T cell response is focused on the bulged region of the bound peptide. Although Glu at P9 lies deep within the groove, it is predicted to stabilize the structure, hence substitution of this residue is likely to radically alter the surface topography of the peptide. Typing of 19 of the T cell clones for expressed TCR Vβ gene subfamilies [33] demonstrated that 2 different subfamilies (Vβ1 and Vβ3) were represented in the clones from both animals (Fig 5). This is consistent with previous data from Vβ analysis of both cultured T cell lines and *ex vivo* CD8^+^ T cells from *T. parva*-immune cattle, which showed that the response to dominant epitopes, although polyclonal, contains a small number of highly abundant clonotypes [20], [33]. However, the results obtained here with the Ala-substituted peptides showed that the Vβ1^+^ and Vβ3^+^ clones each contained different fine specificities, indicating that these clones included different TCR rearrangements, either at the level of the CDR3 sequence or the paired α chain. Hence focusing of the responding T cells on the bulged region of the bound peptide occurred irrespective of the TCR chain rearrangements.

The results shown in Fig 5, using a saturating concentration of peptides (1 ng/ml), reveal the residues that have major effects on recognition by the T cell clones; individual Ala-substitutions at the predicted anchor positions (P5 and 11) had only minor and variable effects on T cell recognition of targets at this peptide concentration. However, substantially reduced recognition relative to the native peptide was observed at lower concentrations, particularly when P11 was substituted (Fig S6). These results indicate that peptides containing single substitutions in the anchor residues retain some MHC-binding activity at high peptide concentrations. Analysis of peptides with multiple Ala-substitutions (P5 and 11, or P2, 5 and 11) demonstrated that these substitutions abolished T cell recognition, consistent with the binding data from MHC stabilization assays.

## Discussion

The cattle pMHC class I structure described here demonstrates that peptides presented by N*01301 are held in an unusual conformation likely to impact significantly on TCR recognition. Binding studies defined the putative anchor positions involved in peptide binding to N*01301 to be P2, P5 and the C-terminal residue. Their relative importance, however, differs depending on length and composition of the peptide. Overall, the binding data suggest that a hydrophobic residue at the C-terminus and an anchor residue Lys at position 5 define the binding motif of N*01301. Functional studies using CD8^+^ T cell clones complement and add to these data, and support the suggestion that the unusual peptide conformation impacts significantly on T cell recognition.

A number of residues within the N*01301 binding groove are involved in the unusual positioning of the 11mer peptide. Position 97 Glu exerts a strong influence on the P5 Lys, pulling it deep into the groove and helping to restrict the bulge in the peptide to its C-terminal end. An anchor at P5, or anywhere in the central region of the peptide, is unusual in human MHC and has only been described with HLA-B *08, B*14, B*15, B*35 and B*51. Of these only B*35 is known to bind unusually long peptides (>10 residues), but even when central anchors are involved the peptide bulge does not take on the conformation shown in the N*01301 structure ([31] Fig. 4A, 4B).

The Trp73 residue is most influential in positioning of the peptide bulge, as described in detail in the *Results* section. While extremely rare in other species, this feature is found in a number of mouse MHC class I alleles, such as H-2D^b^, forming part of a hydrophobic ‘ridge’ that cuts across the peptide binding groove [11]. The Trp73 residue, which contributes to this structural feature, is found only in a small group of closely related cattle class I alleles (Table 1) encoded by class I gene 6 [4], [5]. In the mouse the Trp73 residue is seen in alleles encoded at more than one locus, but Ciatto and colleagues [11] argue for the existence of a ‘related’ family of alleles having a tight evolutionary relationship, possibly with a common progenitor. These authors go on to suggest that the resultant structure of these alleles focuses the conformational variability of the bound peptides in a position that allows optimal TCR interaction, and for this reason propose that evolution has led to conservation of this feature.

Studies of human CTL responses have shown that peptides of between 8 and 10 residues in length constitute the majority of epitopes presented by MHC class I molecules. Recent studies have identified a very small number of longer epitopes that are presented by HLA-B*35 [10], [28], [29]. Typically, non-canonical peptides of >10 amino acids in length bulge from the peptide-binding cleft and there is evidence that this feature can result in biased selection of the TCR repertoire [30], [31]. Results suggest that a rigid bulge in the peptide can lead to selection of a specific T-cell repertoire showing limited diversity, in some instances including public TCR specificities with conserved Vβ and CDR3 sequences. In a number of reported cases the structure is flexible allowing the epitope to be flattened by the TCR interaction (Fig. 4C), providing a more standard landscape; this leads to a diverse TCR profile [31]. The peptide conformation in our structure appears to be relatively rigid and results in an extensive interface area with the binding groove. A likely consequence of such an extensive association is that the strength of this interaction could be substantial and there would be little conformational adaptability of the peptide upon TCR docking, which might imply a smaller energetic penalty upon complex formation with TCRs. We predict that shorter (9 or 10mer) peptides would interact in the same way with the peptide binding groove, with similar impact on T cell recognition.

Data generated using CD8^+^ T cell clones specific for Tp1_214–224_ complement conclusions derived from the structural analysis, and support the suggestion that the unusual positioning of the peptide has a role in the immunodominance of this epitope. Recognition by all 22 CD8^+^ T cell clones studied was disrupted by Ala substitutions in those residues forming the rigid, bulged part of the peptide (P6–P9), with additional variable effects observed with substitutions at other positions, notably P3 and P4. P9 appears to be a particularly critical residue affecting recognition by all T cell clones. Typing of the T cell clones for TCR Vβ gene expression revealed that all 19 clones examined expressed gene segments belonging to the Vβ1 and Vβ3 subfamilies, although the data obtained with Ala-substituted peptides demonstrated different fine specificities among clones expressing the same Vβ gene, indicating different TCR rearrangements. Hence focusing of recognition on the bulged region of the peptide was apparent irrespective of the expressed TCR gene rearrangements of the T cells. The key role played by P9 in influencing T cell recognition is consistent with the structural findings which show this to be the point at which the peptide main chain dips back into the binding groove, making several strong interactions and having a key role in positioning and stability of the bulged section of peptide. Substitution of P9 would therefore be predicted to disrupt both the shape and stability of this region, thus abrogating TCR recognition. The structural data together with the functional results reinforce the crucial nature of peptide conformation in determining the fine specificity of the T cell response.

It is of interest that the N*01301 allele is only found expressed in isolation on the haplotype designated A18. Extensive analysis of animals homozygous for this haplotype indicates that no other classical class I genes are present or expressed [34]. A number of other cattle MHC haplotypes have been identified that also express a single class I gene. Unlike chickens, which have a single, dominantly expressed class I gene [18], such a phenomenon is unusual in mammals, where two or three classical class I genes are usually expressed. The A18 haplotype is very common in British Friesian cattle, which represent a sub-population of the Holstein/Friesian breed and although these animals are subject to artificial selection, it does suggest that this MHC haplotype does not place them at a major immunological disadvantage. Studies of CD8^+^ T cell responses to *T. parva* have demonstrated a hierarchy of dominance in the MHC restriction of the response, with some MHC haplotypes consistently being dominant in preference to others. The dominant restriction elements on these haplotypes include N*01301 and other related alleles shown in Table I, which share the ‘A6’ supertypic class I serological specificity [35] and are predicted to have the hydrophobic ridge likely to result in an unusually positioned peptide. A recent study of CD8^+^ T cell responses to foot and mouth disease virus (FMDV) in cattle also demonstrated that animals carrying the A18 class I haplotype (both homozygotes and heterozygotes) gave a consistently strong N*01301-restricted response to the virus [22]. These data support the hypothesis that this group of alleles demonstrates a common characteristic leading to pMHC/TCR structures that tend to generate dominant CD8^+^ T cell responses.

Despite the antigenic complexity of *T. parva*, the genome of which is predicted to encode >4,000 proteins [36], CD8^+^ T cell responses induced by infection with the parasite show profound immunodominance, in some cases resulting in the majority of the response being focused on a single epitope, as observed with Tp1_214–224_ [20], [35]. Moreover, immunodominance of this epitope was found to be retained in A18^+^ cattle immunized with Tp1 and four additional *T. parva* antigens, delivered by a prime boost protocol in separate vaccine constructs [18]. These findings imply that Tp1_214–224_ presented by N*01301 is inherently highly immunogenic. There is evidence that immunodominance can be influenced at different levels including the generation of an appropriate TCR repertoire, the abundance and turnover of the proteins from which epitopes are derived and the affinity of the peptides for the MHC [15]. Complex pathogens such as *Theileria parva* are likely to generate many abundant peptides capable of binding to a particular MHC class I allele, for which specific T cells are present in the repertoire. Hence there must be additional factors that determine the dominance of a particular epitope. The results shown in the present study indicate that the very prominent C-terminal bulge of the Tp1_214–224_ 11mer when presented by N*01301 and the highly stable nature of the structure, are likely to be key factors in determining the dominance of this epitope over other parasite peptides bound by N*01301.

Immunodominance of CD8^+^ T cell responses to a range of pathogens in human and animal is well-documented and has been shown to result in strain-specificity of responses, allowing escape from immune recognition. Thus the results reported here are not only of fundamental biological interest but also of practical relevance. Defining how diverse peptides bind to different MHC molecules leads to an improved understanding of the structural basis for T cell dependent immune responses. This provides insight into what determines a highly immunogenic peptide-MHC complex and hence can be of value in prediction of antigenic epitopes and vaccine design.

## Materials and Methods

### Identification of a N*01301 Self Peptide

Analysis of peptides bound to the cattle MHC class I allele N*01301 (formerly named HD6) was described in Gaddum et al [14], when a partial binding motif was identified. Subsequently a complete self peptide derived from an eluted peptide pool was sequenced and identified as TIMPKDIQL which is derived from Histone H3.

### Peptide Binding Using Stabilization Assay

RMA-S is a mouse lymphoma cell line with a deficiency in MHC class I antigen processing which has been used extensively to study binding of peptides to MHC class I [37]. RMA-S cells were stably transfected with N*01301 in pcDNA6 and assays performed as described using a mAb (IL-A88, [38]) specific for cattle class I and that recognizes all alleles to detect stable surface expression of N*01301. Peptides were synthesized with fluorethyl-methoxy-carbonyl (fMOC) chemistry (Peptide Protein Research Ltd, Southampton, UK) and purified in-house to >98%. Peptides were used at concentrations between 0.1 nM and 1 mM in steps of 10 fold dilution. Results are expressed as fluorescence index (FI) values. These were calculated as the test mean fluorescence intensity (MFI) minus the no-peptide isotype control MFI divided by the no-peptide N*01301-stained control MFI minus the no-peptide isotype control MFI. The half-maximal binding level (BL_50_), which is the peptide concentration yielding the half-maximal FI of the reference peptide in each assay, was calculated and presented as pBL_50_ (–logBL_50_). The N*01301 self peptide TIMPKLIDQ, which binds with high affinity, was used as a reference peptide.

### Protein Expression, Purification and Complex Assembly

The N*01301 expression construct (amino acids 25–279 of N*01301 plus N-terminal His-tag and C-terminal biotinylation sequence) was generated by ligation-independent cloning (Gateway Technology, Invitrogen) into pOPINI [39], over-expressed in *Escherichia coli* RosettaBlue (DE3) pLacI (Novagen) as inclusion bodies (IB), dissolved in appropriate urea-containing buffers and purified using Ni affinity chromatography. This construct was generated following extensive high-throughput screening using the facilities and expertise of the Oxford Protein Production Facility (www.oppf.ox.ac.uk). Cattle β_2_m cDNA [40] was cloned into pet24a(+) (Novagen), expressed as IB in BL21-CodonPlus (DE3)-RP competent cells (Strategene), and subsequently isolated and dissolved in urea-containing buffers. The denatured proteins were re-natured in the presence of the Tp1_214–224_ peptide for 3 days, concentrated and purified by FPLC (fast protein liquid chromatography) with a HiLoad 26/60 Superdex 75 column (Pharmacia).

### Crystallization and Data Collection

Renatured Tp1_214–224_- N*01301 was concentrated to 6.6 mg/ml in 150 mM NaCl, 20 mM Tris (pH8.5) and crystallized in 20% (wt/vol) PEG 8000 and 50 mM potassium di-hydrogen phosphate using the sitting drop vapor diffusion method. Crystals were briefly transferred to a cryoprotectant solution prior to flash-freezing at 105 K. The crystals diffracted to 1.8 Å resolution; diffraction data were collected at the European Synchrotron Radiation Facility (Grenoble, France) on beamline ID14.2 by using a Quantum 210 charge-coupled device detector (Area Detector Systems, Poway, CA). The X-ray data were processed and scaled with the HKL suite ([41]; Table S1).

### Structure Determination and Analysis

The structure was determined by molecular replacement using HLA-B*5703 (2BVO.pdb) as a search model in AMoRe [42]. Initial rigid body refinement was followed by a series of restrained TLS refinements performed using REFMAC [43] to R_work_ of 19.9% and R_free_ 23.9%. Manual rebuilding was carried out in COOT [44]. Continuous density allowed unambiguous model building of N*01301. No density was visible for the N-terminal His tag or the C-terminal biotinylation signal, which are presumably flexible. Crystallographic statistics for the final models are given in Table S1. All figures of molecular models and electron density were generated in PyMol supplied by Delano Scientific LLC (www.pymol.org) and electrostatic potential was calculated using APBS [45] through the Pymol interface. The atomic coordinates and structure factors are deposited in the Protein Data Bank (www.pdb.org): ID code 2xfx (coordinates) r2xfxsf (structure).

### Analysis of Recognition by CD8^+^ T Cells

CD8^+^ T cell clones specific for Tp1_214–224_ and restricted by N*01301 were derived from 2 cattle, 641 and 633 (MHC class I types A18/A18 and A18/A31), which had been immunized with the Muguga strain of *T. parva* by infection and treatment [46]. The T cell lines were generated by limiting dilution in 96-well plates as described previously [47]. They were expanded and maintained in 48 well culture plates by stimulation at 10–14 day intervals with γ-irradiated autologous parasitized cells in RPMI culture medium containing 10 mM HEPES buffer, supplemented with 10% heat-inactivated fetal bovine serum, 5×10^−5^ M 2-mercaptoethanol, 2 mM L-glutamine, 50 mg/ml penicillin/streptomycin, and 100 units/ml human interleukin-2 (Chiron Corporation, Emeryville, CA., USA). The specificity of the CD8^+^ T cell clones was analyzed using a 4 hour Indium oxide (^111^In, Amersham Medical)-release cytotoxicity assay [47]. Target cells consisted of autologous *T. annulata*-transformed cell lines pulsed with peptides as described by MacHugh et al [20]. The T cell receptor Vβ genes expressed by the T cell clones were identified by PCR analysis of cDNA using bovine Vβ subfamily-specific primers as described by Connelley et al [33].

## Supporting Information

Figure S1 Semi-logarithmic dose/FI curves. The self peptide TIMPKDIQL was used as a reference. High binders have low BL_50_ values (high pBL_50_, pBL_50_ = logBL_50_) and low binders have high BL_50_ values (low pBL_50_). Peptides that did not reach 50% of the binding level of the reference peptide were considered non-binders.
(1.94 MB TIF)

Figure S2 Structural peptide comparisons. Structure comparison of the 11mer peptide Tp1_214–224_(cyan) as bound to N*01301, with peptides bound in various mammalian class I classical MHCs. View shows the Cα-traces of the bound peptide from the α2 helix side of the binding groove, unless stated otherwise.(A) Human allele comparison: HLA-A2 bound 10mer (pink, PBD code 1I4F), HLA-B8 bound 9mer (brown, PBD code 1M05), HLA-B14 bound 9mer (purple, PBD code 3BVN), HLA-B15 bound 9mer (grey, PBD code 3C9N), HLA-B27 bound 9mer (orange, PBD code 2BST), HLA-B35 and 10mer (red, PBD code 2AXG), HLA-B44 bound 10mer (blue, PBD code 3DX9), HLA-B51 bound 9mer (yellow, PBD code 1E27) and HLA-B57 bound 9mer (green, PBD code 2BVP). (B) Human allele comparison: view onto the binding groove of panel A. (C) Species comparison: B21 Chicken bound 11mer (green, PBD code 3BEV), Macaque Mamu-A*01 bound 8mer (pink, PBD code 1ZVS), Rat RT1-A bound 9mer (red, PBD code 1KJM), and Mouse H-2D^d^ bound 10mer (purple, PBD code 1BII). (D) Species comparison: view onto the binding groove of panel C.
(6.33 MB TIF)

Figure S3 N*01301 binding groove - surface representation. Surface representation of the N*0301 binding groove with the Tp1_214–224_ peptide in gold as either (A) a molecular model or (B) a molecular surface. The electrostatic potential on the N*01301 solvent accessible surface has been coloured from blue (electropositive) to white (neutral) to red (electronegative) with increasing color intensity depicting stronger electrostatic potential.
(6.12 MB TIF)

Figure S4 N*01301 binding interactions. Figure shows the binding interactions of the peptide Tp1_214–224_ with the cattle MHC class I molecule N*01301. The classical binding pockets A–F are labeled.
(5.44 MB TIF)

Figure S5 Different aspect views of the N*0301 binding groove in complex with Tp1_214–224_. (A) Cartoon representation of the N*01301 presentation platform in white with the docked Tp1_214–224_ in stick representation colored gold. The view looks down on the presentation platform in the direction that incoming TCRs would attempt docking. Residues of the six pockets have been colored as: pocket A in red, pocket B in green, pocket C in blue, pocket D in magenta, pocket E in brown and pocket F in cyan. (B) and (C) molecular surface representation of the N*01301 allele with the Tp1_214–224_ peptide in stick or surface representations respectively. Different aspect views of the N*01301- Tp1_214–224_ complex in cartoon and space-filling representations. Views (D) looking across the interface from α1 (α1 foreground, α2 background), (E) looking down the binding groove with the N-terminus of the peptide in the foreground, (F) looking across from α2 (α2 foreground, α1 background) and (G) looking down the binding groove with the C-terminus of the peptide in the foreground.
(4.50 MB TIF)

Figure S6 CD8 recognition of Ala-substituted peptides. CD8^+^ T cell recognition of Tp1_214–224_ peptides containing Ala substitutions in predicted anchor sites 2, 5, 11. Data are presented for clone 641.18 tested in a 4-hour ^111^Indium release cytotoxicity assay, using target cells pulsed with the native peptide (solid diamonds) and peptides containing Ala substitutions P11 (solid squares), P5 and P11 (open triangles) and P2, 5 and 11 (open circles).
(0.03 MB DOC)

Table S1 Data collection, phasing, refinement statistics and model quality.
(0.55 MB TIF)

Table S2 Analysis of solvent accessible surface buried in the interface between MHC class I and the peptide presented.
(0.05 MB DOC)
