# Supplementary figures and images for: MHC Class I Bound to an Immunodominant Theileria parva Epitope Demonstrates Unconventional Presentation to T Cell Receptors
Source: PLoS Pathog. 2010 Oct 14;6(10):e1001149. doi: 10.1371/journal.ppat.1001149 (PMC2954893; doi:10.1371/journal.ppat.1001149)

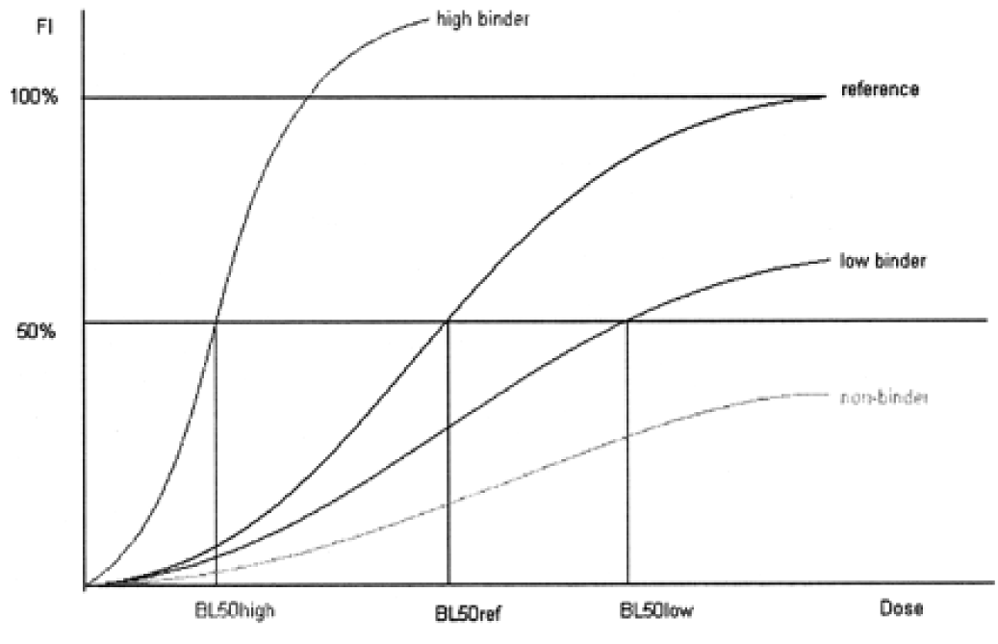

Supplement: Figure S1 — Semi-logarithmic dose/FI curves. The self peptide TIMPKDIQL was used as a reference. High binders have low BL50 values (high pBL50, pBL50 = logBL50) and low binders have high BL50 values (low pBL50). Peptides that did not reach 50% of the binding level of the reference peptide were considered non-binders. (1.94 MB TIF) [file ppat.1001149.s001.tif]

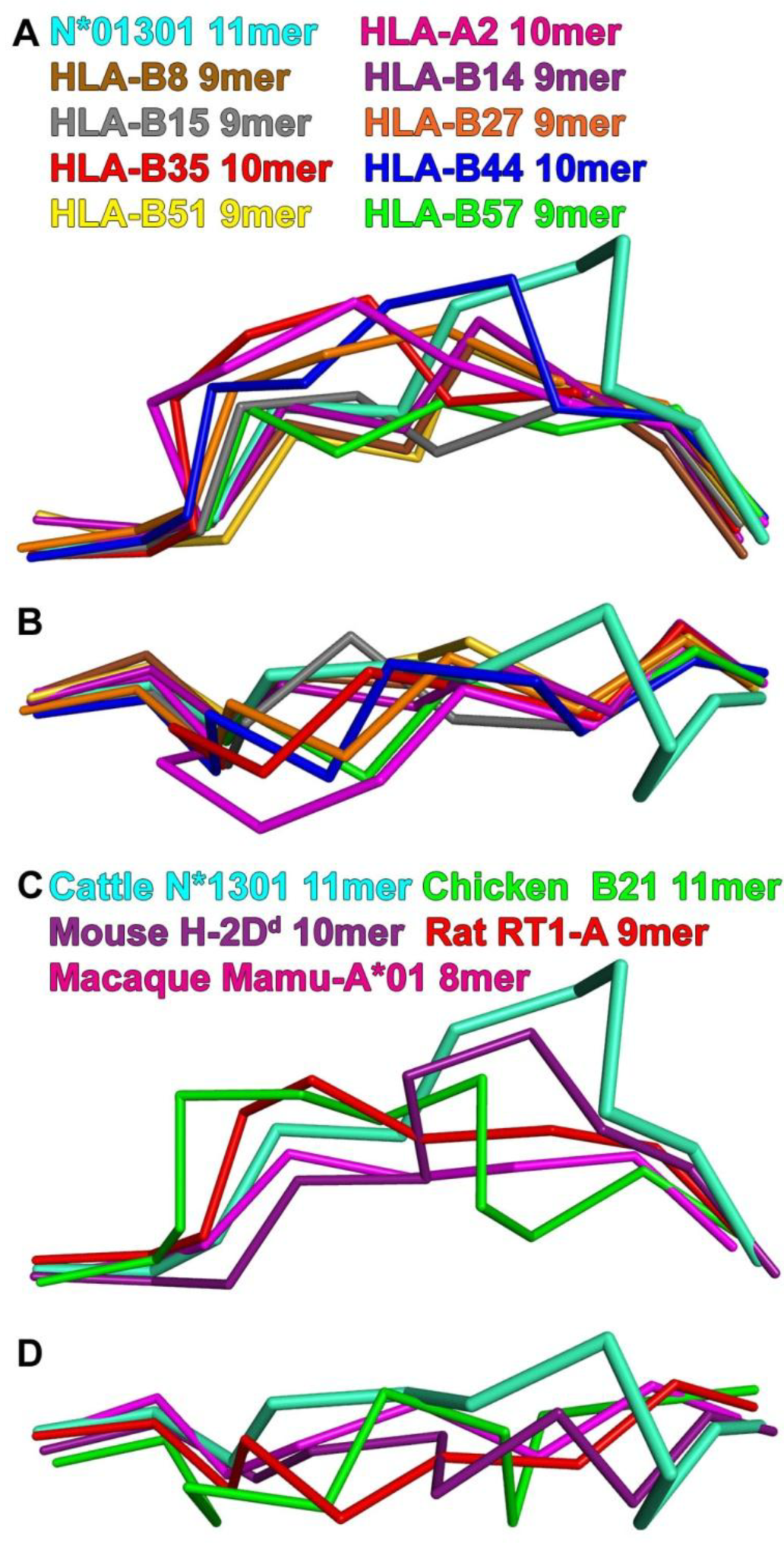

Supplement: Figure S2 — Structural peptide comparisons. Structure comparison of the 11mer peptide Tp1214–224(cyan) as bound to N*01301, with peptides bound in various mammalian class I classical MHCs. View shows the Cα-traces of the bound peptide from the α2 helix side of the binding groove, unless stated otherwise.(A) Human allele comparison: HLA-A2 bound 10mer (pink, PBD code 1I4F), HLA-B8 bound 9mer (brown, PBD code 1M05), HLA-B14 bound 9mer (purple, PBD code 3BVN), HLA-B15 bound 9mer (grey, PBD code 3C9N), HLA-B27 bound 9mer (orange, PBD code 2BST), HLA-B35 and 10mer (red, PBD code 2AXG), HLA-B44 bound 10mer (blue, PBD code 3DX9), HLA-B51 bound 9mer (yellow, PBD code 1E27) and HLA-B57 bound 9mer (green, PBD code 2BVP). (B) Human allele comparison: view onto the binding groove of panel A. (C) Species comparison: B21 Chicken bound 11mer (green, PBD code 3BEV), Macaque Mamu-A*01 bound 8mer (pink, PBD code 1ZVS), Rat RT1-A bound 9mer (red, PBD code 1KJM), and Mouse H-2Dd bound 10mer (purple, PBD code 1BII). (D) Species comparison: view onto the binding groove of panel C. (6.33 MB TIF) [file ppat.1001149.s002.tif]

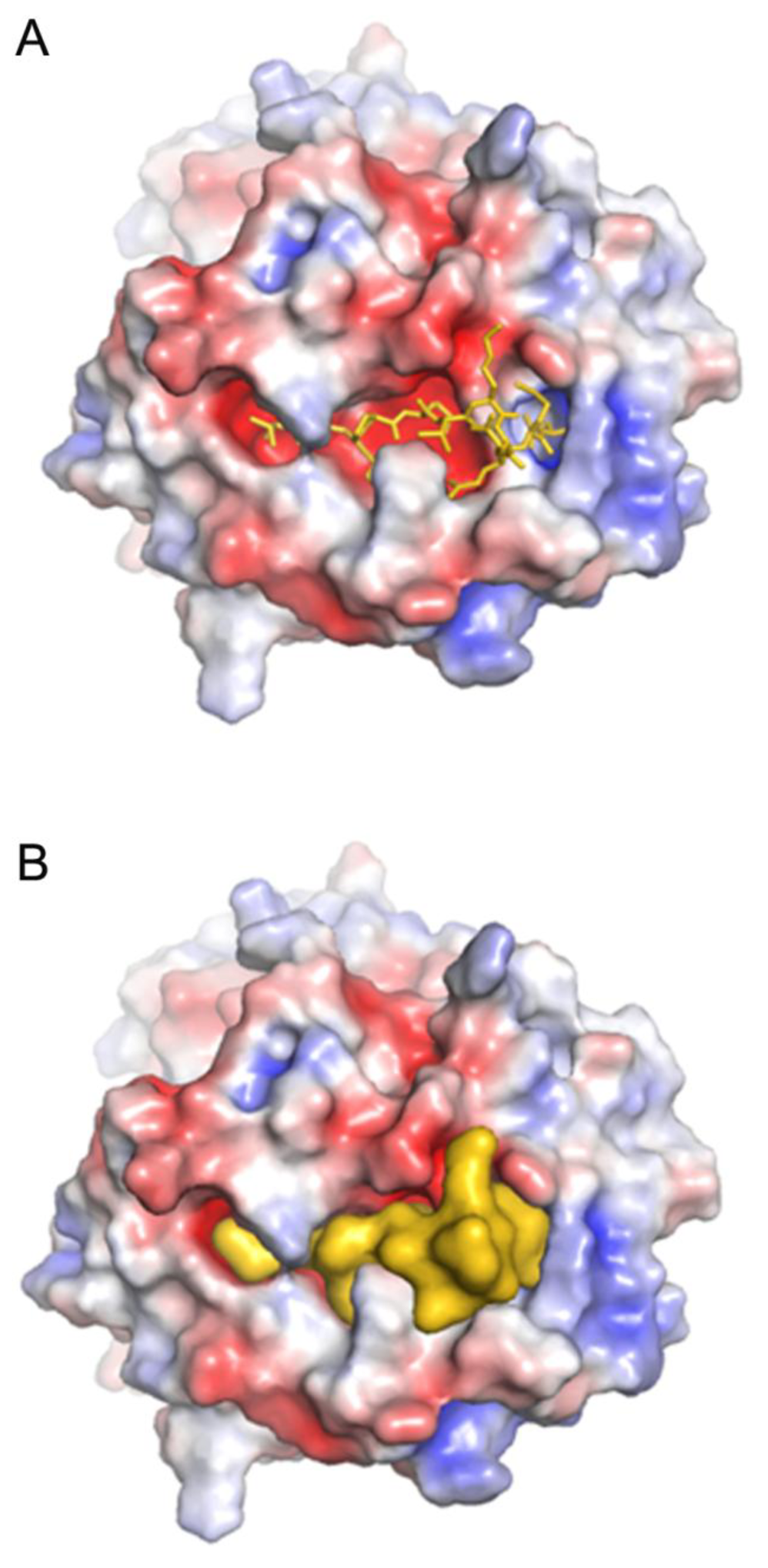

Supplement: Figure S3 — N*01301 binding groove - surface representation. Surface representation of the N*0301 binding groove with the Tp1214–224 peptide in gold as either (A) a molecular model or (B) a molecular surface. The electrostatic potential on the N*01301 solvent accessible surface has been coloured from blue (electropositive) to white (neutral) to red (electronegative) with increasing color intensity depicting stronger electrostatic potential. (6.12 MB TIF) [file ppat.1001149.s003.tif]

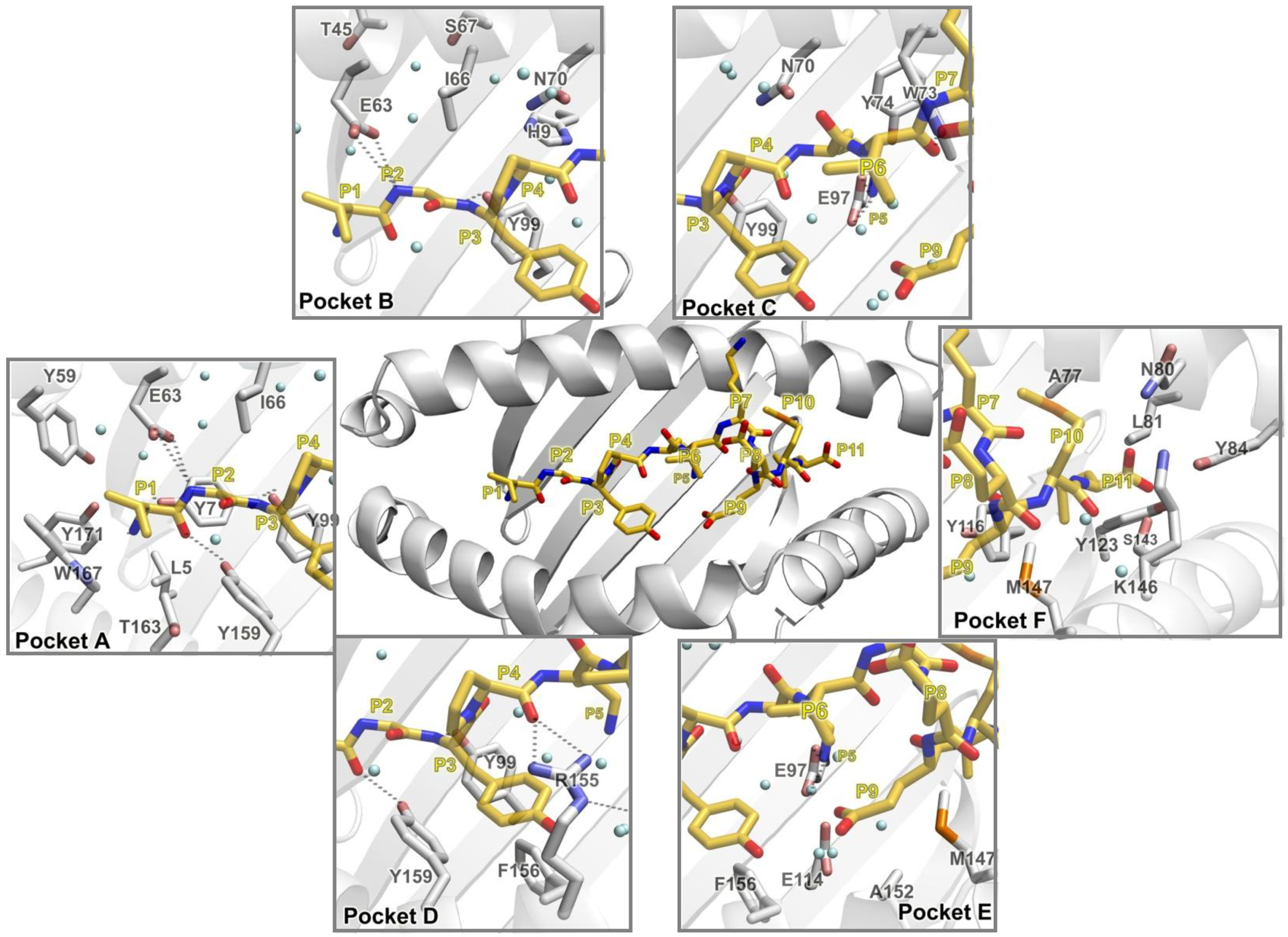

Supplement: Figure S4 — N*01301 binding interactions. Figure shows the binding interactions of the peptide Tp1214–224 with the cattle MHC class I molecule N*01301. The classical binding pockets A–F are labeled. (5.44 MB TIF) [file ppat.1001149.s004.tif]

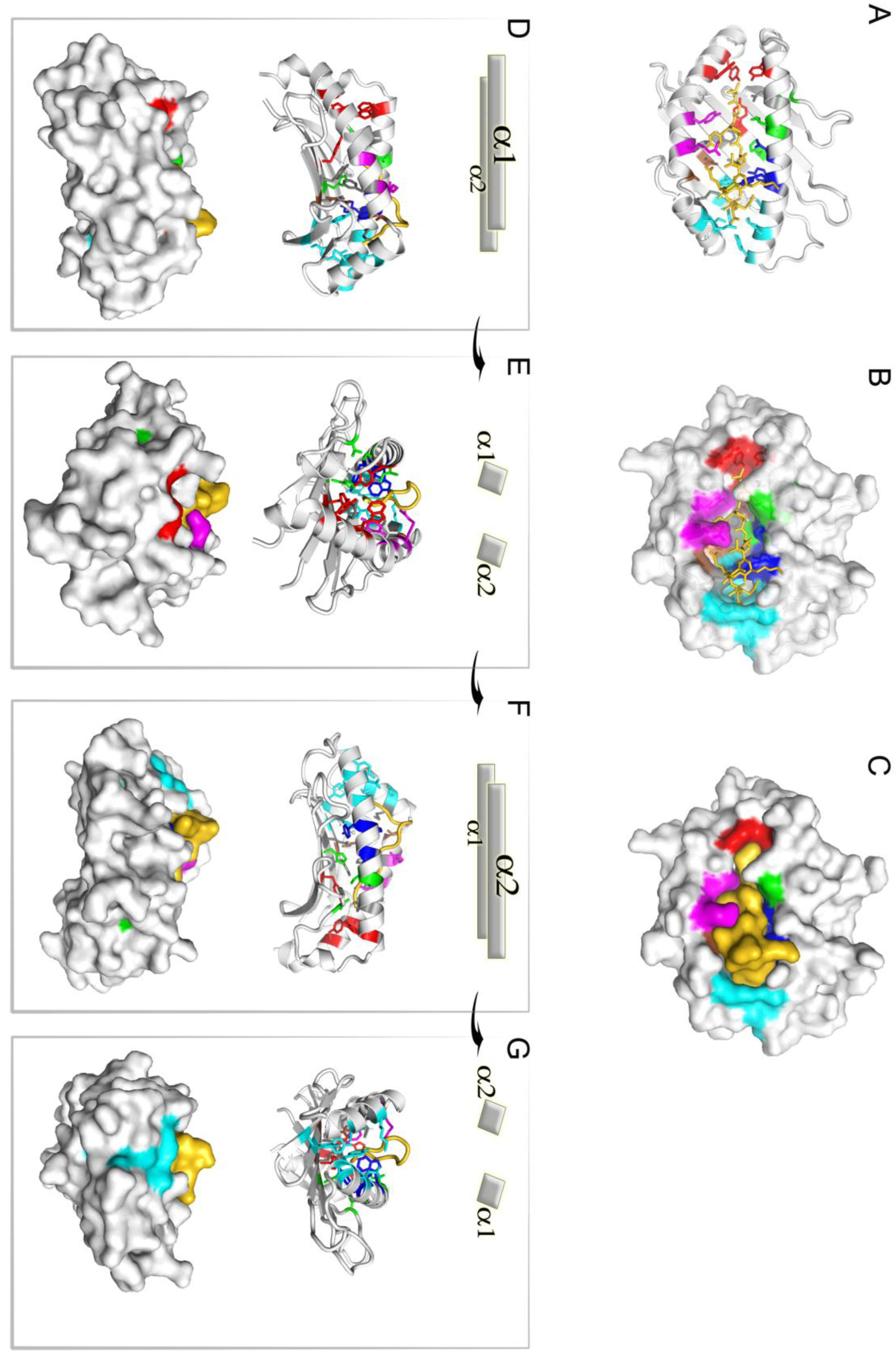

Supplement: Figure S5 — Different aspect views of the N*0301 binding groove in complex with Tp1214–224. (A) Cartoon representation of the N*01301 presentation platform in white with the docked Tp1214–224 in stick representation colored gold. The view looks down on the presentation platform in the direction that incoming TCRs would attempt docking. Residues of the six pockets have been colored as: pocket A in red, pocket B in green, pocket C in blue, pocket D in magenta, pocket E in brown and pocket F in cyan. (B) and (C) molecular surface representation of the N*01301 allele with the Tp1214–224 peptide in stick or surface representations respectively. Different aspect views of the N*01301- Tp1214–224 complex in cartoon and space-filling representations. Views (D) looking across the interface from α1 (α1 foreground, α2 background), (E) looking down the binding groove with the N-terminus of the peptide in the foreground, (F) looking across from α2 (α2 foreground, α1 background) and (G) looking down the binding groove with the C-terminus of the peptide in the foreground. (4.50 MB TIF) [file ppat.1001149.s005.tif]

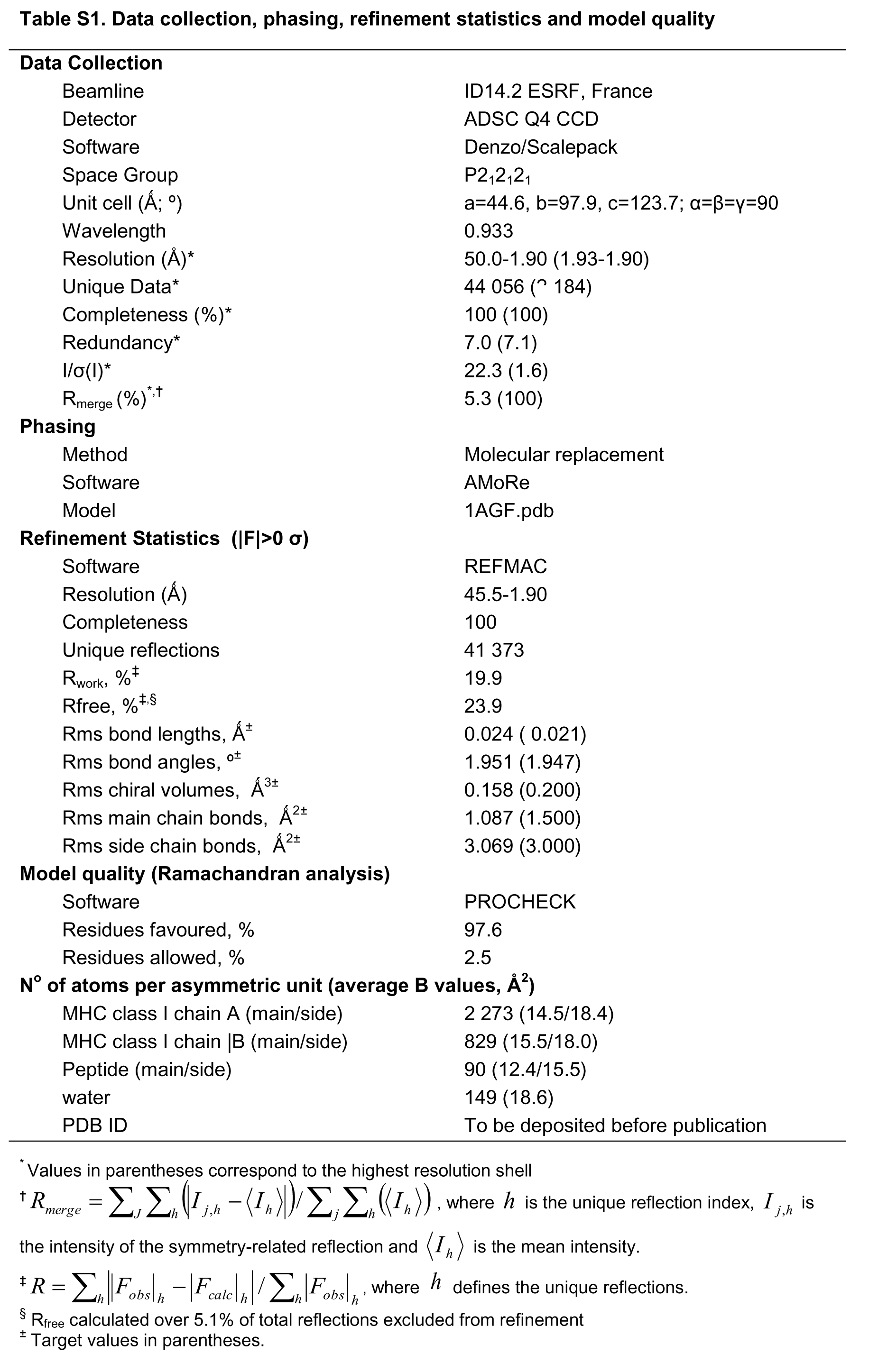

Supplement: Table S1 — Data collection, phasing, refinement statistics and model quality. (0.55 MB TIF) [file ppat.1001149.s007.tif]
