# Supplementary material for: MHC Class I Bound to an Immunodominant Theileria parva Epitope Demonstrates Unconventional Presentation to T Cell Receptors
Source: PLoS Pathog. 2010 Oct 14;6(10):e1001149. doi: 10.1371/journal.ppat.1001149 (PMC2954893; doi:10.1371/journal.ppat.1001149)
Supplement: Table S2 — Analysis of solvent accessible surface buried in the interface between MHC class I and the peptide presented. (0.05 MB DOC) [file ppat.1001149.s008.doc]

**Table S2. Analysis of solvent accessible surface buried in the interface between MHC class I and the peptide presented.**

**PDB Interface Peptide buried Peptide total MHC class I buried surface allele**

**code Area surface surface**

**Ǻ^2^ Ǻ^2^ Ǻ^2^ (ordered res) Ǻ^2^ %**

**1040 1221 1616 (11 res) 858 5.4 N*01301**

----------------------------------------------------------------------------------------------------------------------------------------------------

2h6p 912 1106 1402 (9) 718 4.7 B3501

1a1m 821 977 1320 (9) 665 4.2 B3501

1a1o 967 1129 1424 (9) 809 5.2 B3501

1a9b 861 1021 1357 (9) 700 4.7 B3501

1a9e 871 1057 1354 (9) 685 4.4 B3501

1cg9 838 1035 1374 (9) 642 4.2 B3501

2cik 913 1105 1401 (9) 721 4.7 B3501

2axf 857 1062 1360 (10) 651 4.3 B3508

2axg 843 1030 1331 (10) 656 4.3 B3501

1zsd 920 1131 1576 (11) 709 4.7 B3501

2nw3 934 1155 1587 (11) 713 4.7 B3508

2nx5 906 1529 1077(11) 735 4.7 B3508

2fz3 856 1471 1020(11) 692 4.5 B3508

2fyy 743 914 1207 (7) 572 3.7 B3501

3bw9 945 1123 1560 (12) 767 5.1 B3508

1zhl 931 1157 1765 (12/13) 706 4.7 B3508

1zhk 923 1148 1803 (12/13) 698 4.5 B3501

1xh3 928 1086 1670 (12/14) 769 5.0 B3501

1a1n 856 1021 1252 (8) 691 4.5 B3501

3bwa 793 960 1218 (8) 626 4.1 B3508

----------------------------------------------------------------------------------------------------------------------------------------------------

2rfx 817 968 1274 (9) 667 4.3 B5701

2bvq 808 961 1254(9) 656 4.3 B5703

2bvp 866 1065 1347 (9) 667 4.6 B5703

2bvq 808 961 1254 (9) 656 4.3 B5703

2hjk 867 1002 1617 (11) 732 4.9 B5703

2bvo 834 973 1599 (11) 695 4.7 B5703

2hjl 872 1003 1667 (11) 742 4.9 B5703

----------------------------------------------------------------------------------------------------------------------------------------------------

1m6o 939 1109 1446 (9) 770 5.1 B4402

1n2r 910 1076 1434 (9) 744 4.8 B4403

1sys 943 1132 1494 (9) 755 5.0 B4403

3dx6 924 1086 1605 (9/10) 762 5.0 B4402

3dx7 913 1089 1615 (9/10) 736 4.8 B4403

3dx8 898 1070 1601 (10) 726 4.8 B4405

3dxa 954 1149 1593 (10) 759 4.9 B4405

----------------------------------------------------------------------------------------------------------------------------------------------------

2bst 972 1144 1537 (9) 801 5.2 B2705

2bss 963 1160 1619 (9/10) 766 5.0 B2705

2bsr 990 1162 1675 (9) 818 5.3 B2705

1w0v 1008 1201 1582 (9) 816 5.4 B2705

1jge 816 991 1310 (9) 640 4.1 B2705

1ogt 1067 1287 1846 (9) 847 5.5 B2705

1uxs 1090 1279 1715 (9) 900 5.9 B2705

2a83 1096 1290 1815(9) 901 5.8 B2705

3bp4 931 1142 1431 (9) 719 4.7 B2705

3dtx 935 1138 1138(7) 732 4.8 B2705

1jgd 1003 1209 1682 (10) 797 5.3 B2709

1k5n 801 985 1283 (9) 617 4.1 B2709

1of2 1069 1282 1824 (9) 855 5.6 B2709

1uxw 1041 1236 1828 (9) 846 5.5 B2709

1w0w 1001 1193 1564(9) 810 5.2 B2709

3bp7 949 1152 1427 (9) 745 4.9 B2709

3czf 1081 1277 1823 (9) 884 5.7 B2709

----------------------------------------------------------------------------------------------------------------------------------------------------

1xr8 832 1037 1408 (9) 627 4.1 B1501

1xr9 883 1115 1305 (9) 651 4.3 B1501

3c9n 892 1107 1429 (9) 677 4.5 B1501

----------------------------------------------------------------------------------------------------------------------------------------------------

1e27 865 1038 1329 (9) 693 4.5 B5101

1e28 806 958 1156 (8) 654 4.2 B5101

----------------------------------------------------------------------------------------------------------------------------------------------------

1m05 1004 1214 1496 (9) 794 5.2 B0801

1mi5 986 1164 1477 (9) 807 5.1 B0801

1agb 820 975 1290 (8) 665 4.3 B0801

1agc 812 988 1251 (8) 636 4.3 B0801

1agd 819 989 1275 (8) 649 4.3 B0801

1age 810 975 1296 (8) 645 4.3 B0801

1agf 860 1028 1346 (8) 692 4.6 B0801

----------------------------------------------------------------------------------------------------------------------------------------------------

1akj 857 1039 1332 (9) 675 4.4 A0201

1hhk 868 1025 1424 (9) 710 4.7 A0201

1ao7 838 1010 1453 (9) 665 4.4 A0201

1b0r 635 799 1962 (6) 471 3.2 A0201

1i7r 903 1032 1373 (9) 774 5.0 A0201

1bd2 844 1023 1441 (9) 666 4.5 A0201

1i7t 847 1025 1275 (9) 668 4.5 A0201

1duz 863 1031 1433 (9) 695 4.5 A0201

1i7u 844 1032 1301 (9) 655 4.3 A0201

1akj 857 1039 1332 (9) 675 4.4 A0201

1im3 854 1026 1418 (9) 682 4.6 A0201

1eey 784 956 1333 (9) 613 4.1 A0201

1eez 831 1007 1324 (9) 655 4.4 A0201

1jht 780 968 1242 (9) 593 4.1 A0201

1hhg 763 930 1257 (9) 596 4.0 A0201

1p7q 812 993 1288 (9) 630 4.2 A0201

1hhi 843 1053 1301 (9) 632 4.2 A0201

1qew 840 1003 1380 (9) 676 4.6 A0201

1hhj 847 1025 1359 (9) 669 4.5 A0201

1qr1 830 1011 1274 (9) 650 4.2 A0201

2vll 848 1056 1307 (9) 639 4.3 A0201

2vlk 812 980 1284 (9) 644 4.2 A0201

2vlj 834 1019 1321 (9) 649 4.2 A0201

2v2x 851 1057 1343(9) 644 4.3 A0201

2v2w 832 1004 1318 (9) 660 4.4 A0201

2uwe 809 981 1342 (9) 637 4.2 A0201

1i4f 811 989 1385 (10) 634 4.2 A0201

1jf1 881 1058 1379 (10) 705 4.8 A0201

1hhh 915 1112 1405 (10) 718 4.7 A0201

2gt9 835 1003 1348 (10) 667 4.4 A0201

2clr 896 1086 1477 (10) 705 4.8 A0201

3bh8 947 1116 1577 (10) 777 5.1 A0201

3bh9 886 1050 1490 (10) 721 4.8 A0201

3bhb 972 1143 1598 (10) 801 5.2 A0201

3fqn 711 864 1272 (10) 557 3.7 A0201

3fqr 808 974 1366 (10) 643 4.2 A0201

2jcc 812 978 1332 (9) 647 4.2 A0201

2p5e 762 936 1473 (9) 588 4.0 A0201

2j8u 821 934 1338 (9) 649 4.2 A0201

2guo 757 904 1205 (9) 610 4.1 A0201

2gtz 779 959 1276 (9) 600 4.0 A0201

2pye 795 958 1481 (9) 632 4.1 A0201

2gj6 816 969 1427 (9) 662 4.4 A0201

1qrn 823 1006 1440 (9) 640 4.2 A0201

2p5w 795 962 1469 (9) 628 4.1 A0201

1qse 831 1006 1490 (9) 655 4.4 A0201

1qsf 798 983 1340 (9) 613 4.1 A0201

1s8d 782 972 1294 (9) 591 3.9 A0201

1s9w 904 1086 1463 (9) 723 4.8 A0201

1s9x 870 1042 1425 (9) 698 4.7 A0201

1s9y 882 1061 1438 (9) 702 4.7 A0201

1t1w 852 1065 1397 (9) 639 4.2 A0201

1t1x 838 1044 1375 (9) 632 4.2 A0201

1t1y 845 1039 1363 (9) 651 4.3 A0201

1t1z 808 1008 1303 (9) 608 4.1 A0201

1t20 861 1065 1397 (9) 657 4.3 A0201

1t21 832 1041 1360 (9) 624 4.1 A0201

1t22 845 1042 1346 (9) 648 4.3 A0201

1tvb 826 975 1395 (9) 677 4.6 A0201

1tvh 860 1028 1438 (9) 693 4.6 A0201

2av1 780 967 1416 (9) 632 4.1 A0201

2av7 786 965 1419 (9) 614 4.0 A0201

2c7u 830 991 1327 (9) 668 4.3 A0201

2git 829 996 1364 (9) 661 4.3 A0201

2gtw 751 905 1226 (9) 598 4.0 A0201

2vlr 848 1027 1343 (9) 668 4.2 A0201

3bgm 823 987 1374 (9) 658 4.4 A0201

1jf1 881 1058 1379 (10) 705 4.8 A0201

3d25 827 1015 1414 (9) 640 4.2 A0201

3fqt 711 913 1254 (9) 509 3.4 A0201

3fqu 719 900 1255 (9) 538 3.6 A0201

3fqw 817 991 1253 (9) 643 4.3 A0201

3fqx 833 1005 1319 (9) 660 4.4 A0201

3ft2 754 928 1385 (8) 580 3.8 A0201

3ft3 865 1033 1390 (9) 696 4.6 A0201

3ft4 885 1063 1445 (9) 708 4.7 A0201

3gjf 818 993 1445 (9) 643 4.3 A0201

3hae 727 875 1438 (9) 578 3.8 A0201

1bii 923 1111 1450 (10) 735 4.7 H-2Dd

1ddh 949 1094 1416 (10) 803 5.2 H-2Dd

1qo3 909 1073 1440 (10) 745 4.7 H-2Dd

1ddh 949 1094 1416 (10) 803 5.2 H-2Dd

1wby 884 1100 1554 (10) 668 4.4 H-2Dd

1p42 813 972 1302 (8) 654 4.2 H-2Dd

2clz 933 1074 1283 (8) 792 4.8 H-2Dd

2ve6 904 1098 1414 (9) 711 4.5 H-2Dd

1wbx 958 1170 1548 (10) 746 4.8 H-2Dd

3e6f 852 1019 1318 (9) 685 4.4 H-2Dd

3e6h 878 1069 1386 (10) 687 4.4 H-2Dd

3dmm 914 1094 1448 (10) 733 4.8 H-2Dd

3ecb 910 1058 1412 (10) 762 5.0 H-2Dd

----------------------------------------------------------------------------------------------------------------------------------------------------

1zt1 824 1007 1299 (8) 641 4.0 H-2Kk

1zt7 971 1172 1517 (9) 770 4.8 H-2Kk

----------------------------------------------------------------------------------------------------------------------------------------------------

1ld9 948 1120 1466 (9) 776 5.0 H-2Ld

2e7l 897 1062 1443 (9) 733 7.6 H-2Ld

3e2h 883 1036 1432 (9) 730 7.5 H-2Ld

----------------------------------------------------------------------------------------------------------------------------------------------------

1ce6 861 1040 1306 (9) 683 4.5 H-2Db

1inq 858 1030 1380 (9) 685 4.4 H-2Db

1qlf 872 1060 1339 (9) 685 4.4 H-2Db

2ve6 904 1098 1414 (9) 711 4.5 H-2Db

3cc5 1009 1216 1605 (9) 802 5.2 H-2Db

3cpl 857 1052 1313 (9) 658 4.2 H-2Db

2cii 416 538 734 (4) 294 1.9 H-2Db

1s7u 949 1144 1454 (9) 755 4.7 H-2Db

1s7v 899 1120 1415 (9) 678 4.5 H-2Db

1s7w 978 1179 1478 (9) 777 4.8 H-2Db

1s7x 937 1141 1440 (9) 733 4.5 H-2Db

1n5a 942 1152 1451 (9) 733 4.6 H-2Db

3ch1 935 1167 1537 (9) 703 4.4 H-2Db

3cch 903 1076 1466 (9) 731 4.6 H-2Db

2zok 894 1095 1420 (9) 693 4.7 H-2Db

2zol 849 1046 1288 (9) 653 4.4 H-2Db

2vlj 834 1019 1321 (9) 649 4.2 H-2Db

2vlk 812 980 1284 (9) 644 4.2 H-2Db

2vll 848 1056 1307 (9) 639 4.3 H-2Db

2vlr 848 1027 1343 (9) 668 4.2 H-2Db

2v2w 832 1004 1318 (9) 660 4.4 H-2Db

2v2x 851 1057 1343 (9) 644 4.3 H-2Db

2j8u 821 994 1338 (9) 649 4.2 H-2Db

----------------------------------------------------------------------------------------------------------------------------------------------------

1vgk 912 1133 1357 (9) 690 4.6 H-2Kd

----------------------------------------------------------------------------------------------------------------------------------------------------

1fo0 877 1011 1284 (8) 742 4.7 H-2Kb

1kj2 891 1050 1318 (8) 733 4.6 H-2Kb

1kj3 907 1069 1325 (8) 745 4.7 H-2Kb

1mwa 850 972 1310 (8) 728 4.7 H-2Kb

1osz 922 1089 1345 (8) 756 4.9 H-2Kb

1p4l 813 972 1302 (8) 654 4.2 H-2Kb

1vac 880 1031 1298 (8) 729 4.8 H-2Kb

1vad 854 991 1314 (9) 717 4.7 H-2Kb

2ol3 869 1019 1336 (8) 719 4.6 H-2Kb

2vaa 905 1035 1305 (8) 775 5.1 H-2Kb

2vab 815 935 1275 (9) 695 4.5 H-2Kb

1bqh 892 1064 1286 (8) 720 4.6 H-2Kb

3c8k 860 1017 1285 (8) 703 4.5 H-2Kb

2fo4 797 918 1199 (8) 676 4.4 H-2Kb

1rk0 814 955 1216 (8) 674 4.4 H-2Kb

1rk1 826 974 1230 (8) 677 4.5 H-2Kb

1s7q 830 1001 1210 (8) 659 4.2 H-2Kb

1s7r 944 1095 1414 (9) 733 5.1 H-2Kb

1s7s 865 1040 1251 (8) 690 4.4 H-2Kb

1wbz 831 973 1296 (9) 689 4.4 H-2Kb

1s7t 845 999 1199 (8) 688 4.4 H-2Kb

1lk2 792 939 1204 (8) 645 4.2 H-2Kb

1p1z 831 962 1244 (8) 699 4.6 H-2Kb

1p4l 813 972 1302 (8) 654 4.2 H-2Kb

1kpu 897 1035 1314 (8) 758 4.8 H-2Kb

1kpv 809 945 1259 (9) 674 4.3 H-2Kb

1nam 914 1071 1308 (8) 757 4.7 H-2Kb

1nan 918 1056 1292 (8) 780 4.9 H-2Kb

1mwa 871 1000 1398 (9) 742 4.8 H-2Kb

1lek 857 1007 1368 (8) 706 4.7 H-2Kb

1leg 870 1023 1324 (8) 718 4.8 H-2Kb

1kj2 887 1036 1314 (8) 738 4.7 H-2Kb

1kj3 903 1063 1307 (8) 743 4.7 H-2Kb

1fo0 877 1011 1284 (8) 743 4.7 H-2Kb

1osz 922 1089 1345 (8) 756 4.9 H-2Kb

2mha 870 994 1319 (8) 747 4.7 H-2Kb

----------------------------------------------------------------------------------------------------------------------------------------------------

1kjm 837 1034 1290 (9) 640 4.1 Rat

----------------------------------------------------------------------------------------------------------------------------------------------------

1zvs 743 926 1167 (8) 559 3.6 Monkey

----------------------------------------------------------------------------------------------------------------------------------------------------

3bev 897 1087 1475 (11) 708 4.5 Chicken

3bew 963 1121 1416 (10) 804 5.2 Chicken

All calculations performed with PISA (Protein interfaces, surfaces and assemblies) service at European Bioinformatics Institute.
