# Supplementary material for: MHC Class I Bound to an Immunodominant Theileria parva Epitope Demonstrates Unconventional Presentation to T Cell Receptors
Source: PLoS Pathog. 2010 Oct 14;6(10):e1001149. doi: 10.1371/journal.ppat.1001149 (PMC2954893; doi:10.1371/journal.ppat.1001149)
Supplement: Supplementary file 3 [file ppat.1001149.s006.doc]

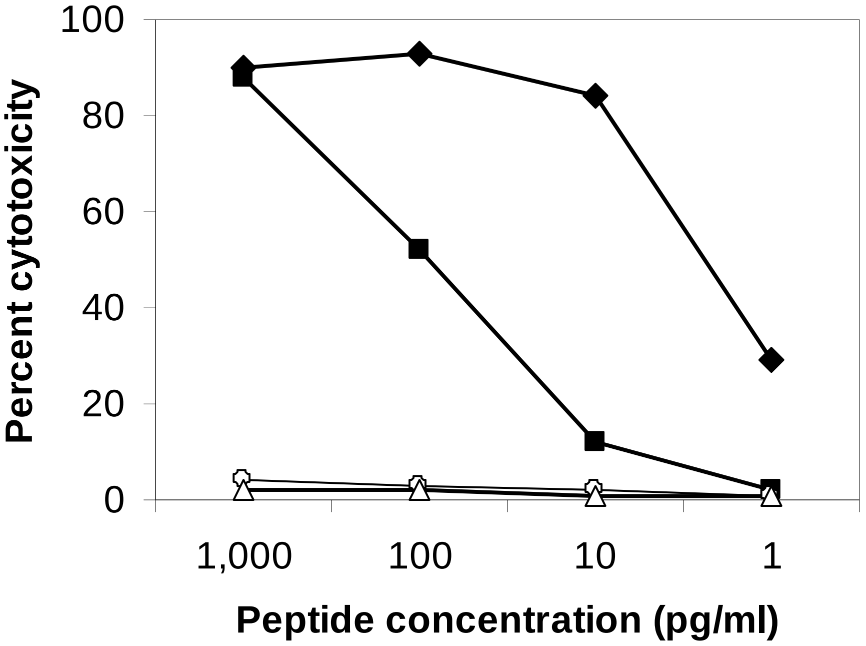
100
80
60
40
20
0
Percent cytotoxicity
1,000
100
10
1
Peptide concentration (pg/ml)
